# Resource Management Scheme Based on Ubiquitous Data Analysis

**DOI:** 10.1155/2014/156083

**Published:** 2014-08-13

**Authors:** Heung Ki Lee, Jaehee Jung, Gangman Yi

**Affiliations:** ^1^Samsung Electronic Co., Suwon, Republic of Korea; ^2^Department of Computer Science & Engineering, Gangneung-Wonju National University, Gangwon-do, Republic of Korea

## Abstract

Resource management of the main memory and process handler is critical to enhancing the system performance of a web server. Owing to the transaction delay time that affects incoming requests from web clients, web server systems utilize several web processes to anticipate future requests. This procedure is able to decrease the web generation time because there are enough processes to handle the incoming requests from web browsers. However, inefficient process management results in low service quality for the web server system. Proper pregenerated process mechanisms are required for dealing with the clients' requests. Unfortunately, it is difficult to predict how many requests a web server system is going to receive. If a web server system builds too many web processes, it wastes a considerable amount of memory space, and thus performance is reduced. We propose an adaptive web process manager scheme based on the analysis of web log mining. In the proposed scheme, the number of web processes is controlled through prediction of incoming requests, and accordingly, the web process management scheme consumes the least possible web transaction resources. In experiments, real web trace data were used to prove the improved performance of the proposed scheme.

## 1. Introduction

Ubiquitous personal devices such as notebooks, smartphones, and web-enabled televisions enable their users access to the internet at any time. Further, people can access their preferred online media, such as social networking sites, on the go. This convenience in access to web services has led to an increase in web traffic. Frequent accesses to a web service result in a heavy burden on the web server, which in turn may result in delays in processing users' requests. Long response times for incoming requests decrease the web server's quality of service. Web users may even cancel their requests. To protect service quality, service providers should provide the requested web documents to web users as soon as possible. To address this issue, web service providers have built web cluster servers for improving web throughput; however, meeting users' demands remains a challenging task.

In general, a web document includes several data from the server, including; html, images, audio, and video. To retrieve a single web document for a user, the web browser obtains one main web object and several related, embedded web objects of the requested web document. The web browser detects the main web object to retrieve the list of embedded web objects for the requested web document. Based on the list of embedded web objects, the web browser sends requests to retrieve them. When the retrieval of one embedded web object is delayed, the overall transaction time for the web document is increased. Therefore, in order to view a web document, web user needs to wait till all the embedded web objects are retrieved.

A web server system must manage limited resources to process incoming requests in a relatively brief time. It is critical to manage the number of idle web processes for future requests. If the web server system only creates processes reactively, that is, after receiving requests from a user, it takes more time. To reduce the time required to generate processes, the web server system proactively creates idle web processes. When a web browser establishes a new connection with the web server system, idle processes are assigned to process requests from the web browser. Such idle processes reduce the processing time of incoming requests. However, maintaining many idle processes will waste resources, including CPU time and memory. Therefore, an efficient web process management scheme is needed to maintain the proper number of processes for future requests.

To determine a suitable number of idle processes, we need to predict how many requests may arrive from web browsers. In a single web document, there is one main object and several embedded objects, such as images, audio, and video. Therefore, the web browser sends requests for both the main object and embedded objects to the web server in order to retrieve one web document. First, the web browser requests the main object from the web server. The web browser establishes one TCP connection with the web server, and the server assigns one idle process to handle this incoming request. After retrieving the main object, the web browser extracts a list of embedded objects based on the main object. Finally, the browser sends requests for the embedded objects. In a modern web framework, the web browser establishes several TCP connections with the web server to retrieve embedded objects in parallel. The web server assigns idle processes to retrieve the embedded objects. Unfortunately, it is impossible to predict when a web browser will establish a new TCP connection to obtain the main web object. It is also difficult to predict how many requests for embedded objects will follow the initial request for the main object. Because of the web cache system, some requests for embedded web objects after the main web object are unnecessary to send to the web server system. When a web cache system in a local area or proxy server already contains requested web objects, it will provide the cached web objects directly to the web browser. Such requests for web objects retrieved from the web cache system are not sent to the web server system. Dynamic access patterns from the web cache system help determine the required number of idle processes.

**Figure 1:**
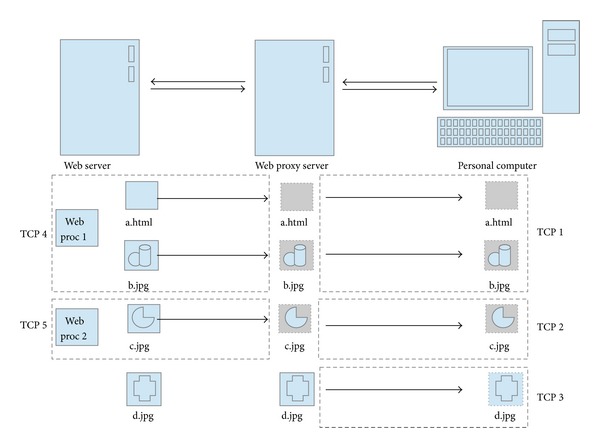
Retrieval of web document.

In Figure 1, the web browser displays a web document, “a.html” to the web user. First, the web browser sends requests to the web proxy server, but the proxy server forwards the requests for “a.html” to the web server after checking whether the requested document is contained in the local area. The web server assigns one process “web proc 1” to handle the request from the web browser. The web proxy server caches the requested web document “a.html” on its own local area and then forwards the web document “a.html” to the web browser. The web client ascertains the list of embedded objects related to “a.html,” including “b.jpg,” “c.jpg,” and “d.jpg.” The web browser establishes two more TCP connections to the three embedded web objects in parallel. One connection has already been established for retrieving “a.html”; therefore, the web browser reuses the already established connection to retrieve “b.jpg.” In addition, the web browser establishes two more connections with the web proxy server to retrieve “c.jpg” and “d.jpg.” To retrieve “b.jpg” and “c.jpg” the web proxy server establishes one more connection with the web server. The web proxy server also reuses the previous connection to retrieve “b.jpg.” The web server reuses “web proc 1” to retrieve “b.jpg” and assigns the new “web proc 2” to retrieve “c.jpg.” After retrieving “b.jpg” and “c.jpg” from the web server, the proxy server caches “b.jpg” and “c.jpg” and sends them back to the web browser as “a.html.” However, “d.jpg” is already cached at the web proxy server. The request for “d.jpg” is answered by the web proxy server and not the web server. The web browser establishes a different number of TCP connections with the web server depending on the web cache system. As a result, a different number of processes are assigned by the web server in response to the web browser. When the web browser requires more embedded objects, the web browser increases the number of TCP connections to the web server.

In this paper, we propose a process management scheme called* ConWebPro*, based on our analysis of the structure of a web document. To predict the number of requested TCP connections from web browsers,* ConWebPro* calibrates the relationship between web objects. A web browser establishes several concurrent web connections to retrieve embedded web objects. Usually, the web browser increases the number of web connections to increase the throughput of the web server system. The web server system should maintain enough idle processes to process requests for embedded objects.* ConWebPro* adjusts the number of idle processes to handle incoming requests for embedded objects after providing the web main object to the browser. When a browser requests main web objects that include several embedded objects,* ConWebPro* increases the number of idle processes. When main web objects contain only a few embedded web objects,* ConWebPro* maintains the current number of idle web processes. We suggest that* ConWebPro* avoids the waste of web system resources caused by maintaining an excessive number of idle processes.

The remainder of this paper is organized as follows.  Section 2 describes our motivation and related work aimed at improving the performance of web server systems.  Section 3 discusses the details of our process management scheme. We present the result of our prediction scheme using real web workloads and the results of a simulation in Section 4.  Section 5 presents our conclusions.

## 2. Related Works

General web server systems like Apache assign one process to handle incoming requests. The process reads documents or obtains the information from a database depending on the requests from web browsers. Some web management schemes improve the performance of web server systems by using extra system resources. Therefore, it is critical to assign resources efficiently to improve the performance of a web server system. CPU time and memory are essential resources to consider when deciding the transaction time. If numerous idle processes are running concurrently to process future requests, the server can save the time it would otherwise take to generate process to handle incoming requests. However, such a scheme also consumes resources.

### 2.1. Background

#### 2.1.1. Persistent Connection and Web Pipeline Scheme

To improve the quality of the web service, most web browsers provide persistent connection and pipeline schemes. Persistent connection and pipeline schemes consume web server system resources to reduce the time spent on inefficient transactions such as generating processes or obtaining web objects sequentially.

**Figure 2:**
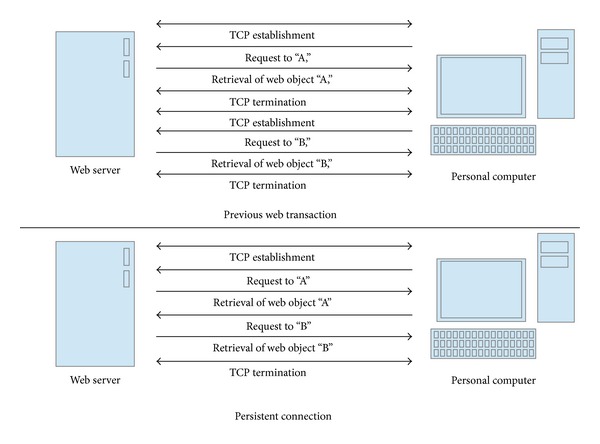
Retrieval of web document by the single connection.

Through a persistent connection scheme, the web browser obtains several web objects from the same connection. In Figure 2, the web browser acquires two web objects, including object “A” and object “B.” In the previous scheme, the browser must establish a new connection with the server to retrieve each object. After retrieving web object “A” the browser terminates the connection with the server. Subsequently, the browser establishes a new connection with the server to retrieve web object “B.” Processes and connections to handle previous requests are reused for future requests from the same web browser. It can save transaction time to reestablish the connection and process, but it wastes memory to maintain processes dedicated to specific web browsers.

**Figure 3:**
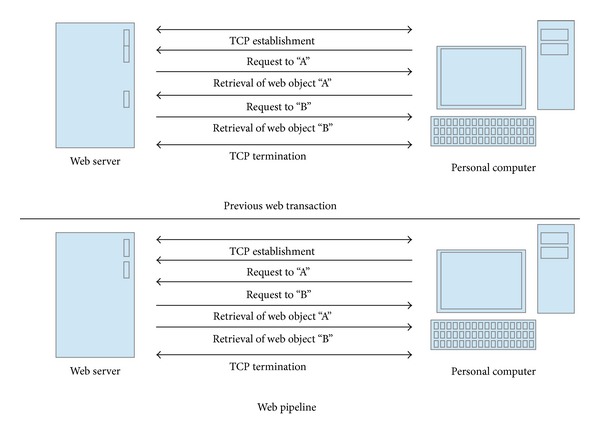
Retrieval of web document by the multiple connection.

Web browsers can also save transaction time using a web pipeline scheme. A web pipeline scheme enables a web browser to retrieve related web objects simultaneously.  Figure 3 shows the difference between the previous scheme and a web pipeline scheme. In the previous scheme, the web browser sends requests to the next web object after retrieving the current requested web object. However, a web browser using the web pipeline scheme sends its request to the next web object before retrieving current requested web object. If the web browser establishes only one connection with a web server, this scheme cannot significantly decrease the transaction time. However, current web browsers utilize multiple connections with servers. A web browser sends several requests concurrently through multiple connections. When a web browser establishes multiple connections, the web pipeline scheme improves the performance of the web server system.

Persistent connection and web pipeline schemes enhance the performance of web servers while also requiring limited system resources.

### 2.2. Dynamic Access Patterns

Within the structure of a web object, a web browser accesses the related embedded objects after accessing the main object. However, the incoming access pattern for web objects is dynamic. When a web browser sends requests for embedded objects, some of the embedded objects are provided by a proxy server, rather than the web server. Proxy servers hold web objects after retrieving them from a web server. When these objects are requested again, the proxy server provides them to web browsers. Requests for the cached web objects are therefore not transmitted to the web server, thus releasing overhead. However, such a cache mechanism makes it difficult for the web server to determine how many requests are incoming from browsers. In Table 1, we see which embedded objects are requested after requesting one main object, “/atomicbk/main.html” at ClarkNet web traces from [1, 2]. When the main object is requested, “orders.gif” is requested most often. However, “stats.gif,” “userlink.gif,” and “comic75.gif” are not requested frequently after retrieving main object.

### 2.3. Previous Works

There have been many previous attempts to predict incoming user requests. We classify examples here into two categories: chaining schemes based on Markov models and grouping schemes based on clusters of web objects.

Chaining schemes are based on *n*th Markov models. A high order of Markov model can provide more accurate predictions; however, increasing the order also increases the complexity of prediction scheme. As a result, chaining schemes restrict the order of the Markov model. Some schemes, such as [3–6] use top-*n* related objects to predict the next requests. Other schemes, such as [4, 7–10] use long access sequences. The work in [11] designs dynamic P.P.M. models.

Grouping schemes generates clusters of web objects and then predicts a group of web objects for the next incoming requests. The work in [12–14] provides a caching policy for a Content Distribution Network platform. The work in [15, 16] designs a caching policy for mobile environments. The work in [17] provides a prediction scheme using folder structure. [18] suggests a divide-and-merge scheme via a hybrid of top-down and bottom-up schemes. The work in [19] designs a proxy model for prefetching embedded objects. The work in [20, 21] uses a vector model and semantic power for a web cluster system.

Hybrid schemes design a prediction scheme based on both Markov models and grouping schemes. The work in [22, 23] suggests prediction schemes based on several concurrent models, including Markov models, association rules, and grouping schemes. The work in [24] uses an abstraction scheme for defining access patterns and defines user access paths through a Markov model. The work in [25] generates a group of access patterns to web objects using a K-means cluster scheme.

Although many examples of research provide prediction schemes for incoming requests, they do not provide process management schemes for modern web frameworks.

## 3. Web Process Management through Structure of Web Document

### 3.1. Prediction Scheme

The work in [1] designs a web transaction prediction scheme called the Double PPM Scheme (DPS). When a web browser requests the main object, the browser also sends requests for related embedded objects to the server. Therefore, we can create a prediction scheme based on a grouping of one main web object and its related embedded objects.

**Figure 4:**
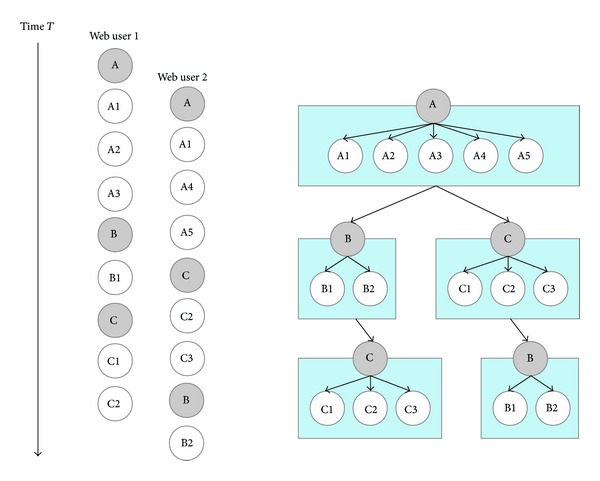
Double P.P.M. scheme.

In Figure 4, DPS predicts the relationship between web objects in several steps. In the first step, DPS distinguishes between main and embedded objects, classifying objects based on their name. For example, when the name of an object includes “html,” “php,” or “jsp,” it is recognized as a main object; usually such objects are web documents. However, when the name of an object contains “jpg,” “mpg,” or “ogg,” they are classified as an embedded object. In the second step, DPS creates relationships between objects. Gray circles in Figure 4 indicate main objects, while white circles indicate related embedded objects. Arrows between circles show how frequently two web objects are accessed together in a single session. These symbols show the relationships within a single web document.

Continuing in Figure 4, we see that this instance of DPS creates three groups: “A,” “B,” and “C.” There are two different access patterns in this log file. After accessing document “A,” some users access document “B” followed by document “C,” whereas other users access document “C” followed by document “B.”

### 3.2. Web Process Management

*ConWebPro* determines the number of web processes to run based on the access patterns of web objects. DPS finds different access patterns based on the structure of these objects. If one document contains many embedded objects, a web browser should access the server after accessing the main object. Similarly, when a requested web document includes dynamic or frequently changed objects, browsers create more HTTP connections to obtain these related embedded objects.

**Figure 5:**
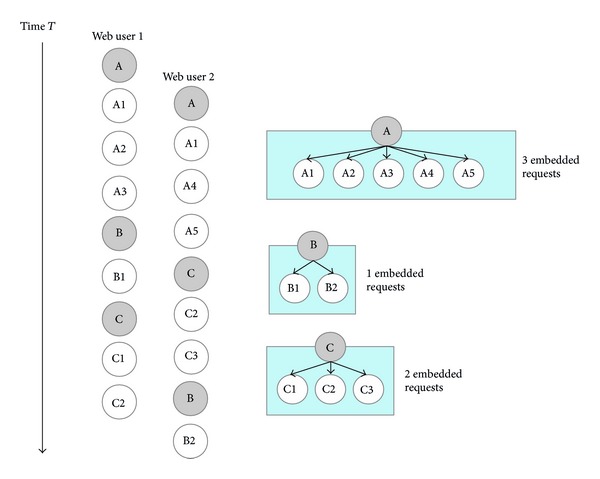
Classification of web objects depending on related embedded objects.

For analysis of web log mining,* ConWebPro* determines how many requests to embedded objects are incoming after retrieving the main object. In Figure 5,* ConWebPro* forms three groups depending on the number of related embedded objects. For document “A,” even though “A” contains five embedded objects, three embedded objects are requested after retrieving the main object. Document “B” has two embedded objects and document “C” contains three embedded objects, respectively. However, when web users request document “B” or document “C,” the browser issues one or two requests for embedded objects.

If the main object is requested,* ConWebPro* anticipates how many requests for embedded object are forthcoming. If document “A” is requested, three requests will come to the server. Therefore, the server system creates three more processes to handle these incoming requests.

When processing requests from browsers, the server system assigns processes to handle incoming requests. It also takes time to process these requests

(1) $$\begin{array}{c} T_{\mathrm{trans}}=T_{\text{init}}+T_{\text{proc}}, \end{array}$$

where *T*_trans⁡_, *T*_init_, and *T*_proc_ are whole transaction time, initial delay in preparing process, and response time, respectively. *T*_init_ can be different depending on the number of idle processes. When an idle process is already generated, we can save transaction time. Accordingly, we obtain (2) from (1):

(2) $$\begin{array}{c} T_{\mathrm{trans}}=H*T_{\text{ini}{\text{t}}_{\text{hit}}}+F*T_{\text{ini}{\text{t}}_{\text{fail}}}+T_{\text{proc}}, \end{array}$$

where *H*, *T*_init_hit__, *F*, and *T*_init_fail__ are hit rate, initial delay with idle process, fail rate, and initial delay without idle process. Accordingly, we obtain (3), because there is no initial delay at *T*_init_hit__:

(3) $$\begin{array}{c} T_{\mathrm{trans}}=F*T_{\text{ini}{\text{t}}_{\text{fail}}}+T_{\text{proc}}, \end{array}$$

*F*, *T*_init_fail__, and *T*_proc_ are determined depending on the number of idle processes. If idle processes are increased, *F* is decreased but *T*_init_fail__ and *T*_proc_ are increased. Too many running processes will waste the limited resources of the web server system. As a consequence, the overall performance of web server system will be degraded:

(4) $$\begin{array}{c} \text{N}{\text{P}}_{\text{idle}}\propto \frac{1}{F}\propto T_{\text{ini}{\text{t}}_{\text{fail}}}\propto T_{\text{proc}}, \end{array}$$

where NP_idle_ is the number of idle processes awaiting future requests. Based on the equation above, a server system should run a small number of idle processes that do not increase *F* for decreasing transaction time. To determine the proper number of idle processes, we should predict future web traffic. Unfortunately, such a prediction is not easy. Overall requests are classified into two groups including requests to main web objects and requests to embedded web objects:

(5) $$\begin{array}{c} \lambda_{\text{total}}=\lambda_{\text{main}}+\lambda_{\text{embedded}}, \end{array}$$

where *λ*_total_, *λ*_main_, and *λ*_embedded_ are overall requests, requests to main objects, and requests to embedded objects, respectively. It is not easy to predict *λ*_total_ and *λ*_main_ at web server, because web user can access web document at any time. When user accesses web document, web browser starts to send request to main object of these document. However, we can predict requests for embedded objects, *λ*_embedded_, through DPS. In* ConWebPro*, we increase the hit rate of idle process through the DPS scheme.* ConWebPro* increases the performance of web server with accurate predictions.

## 4. Performance Evaluation

### 4.1. Simulated System Configuration

We demonstrated the performance of* ConWebPro* by applying it to real web traces collected over the course of two days. Based on DPS in [1], we obtained relationships between web objects using the first day of web traces.* ConWebPro* then classified web documents into three groups depending on the number of embedded objects.  Table 2 shows real traces from web sites including the Department of Computer Science in Gangneung-Wonju National University, NASA, and ClarkNet from [1].* ConWebPro* obtained relationship between web objects based on Day 1. Based on the results,* ConWebPro* created web processes on Day 2. Web browsers retrieved objects through persistent connections and pipelines on HTTP 1.1. Therefore, the browsers retrieved multiple embedded objects simultaneously.

### 4.2. Evaluation Results

Evaluating the performance of* ConWebPro*, two schemes were compared on an Apache web server. An Apache server typically maintains a static number of idle processes to handle incoming requests from web users. This inefficient process management scheme wastes the available memory of the server, causing its performance to drop.

#### 4.2.1. Step  1: Analysis on Web Objects

*ConWebPro* obtains the structure of web objects based on the web log file from Day 1. As its first step,* ConWebPro* forms groups of web requests from the same users. This web log file contains all of the requests from every web user. If multiple users access the web server simultaneously, it is difficult to detect the relationship between objects.* ConWebPro* extracts requests from the same user based on the client IP address found in the web log.

As its second step,* ConWebPro* classifies web objects into main objects and embedded objects through path and access time. In general, web users request “html” or “script” documents from the web server.* ConWebPro* classifies “html” and “script” documents as main web objects. In addition, embedded web objects are requested simultaneously after requesting main web objects.* ConWebPro* makes a group that contains requested main object and related embedded objects simultaneously.

As its final step,* ConWebPro* determines a relationship based on frequency of requests for web objects. After each request for a main web object,* ConWebPro* checks which embedded web objects are accessed frequently.  Table 3 shows 10 main web objects and related embedded web objects at Gangneung-Wonju National University. Each main web object contains approximately 10 embedded web objects. Tables 4 and 5 show 10 main web objects and related embedded web objects from the ClarkNet and NASA web traces.

#### 4.2.2. Step  2: Number of Web Processes

The web log file does not indicate when a web client disconnects from the web server. We are assuming that the web client ends its connection with the web server after retrieving web documents including one main object and multiple embedded objects. The web server can also reorganize the number of processes after releasing connections. As with general web servers, the static scheme attempts to maintain a predefined number of processes.* Static-2* creates two processes, while* Static-10* creates 10 processes for incoming requests. Our* ConWebPro* creates two processes for incoming main web objects.

**Figure 6:**
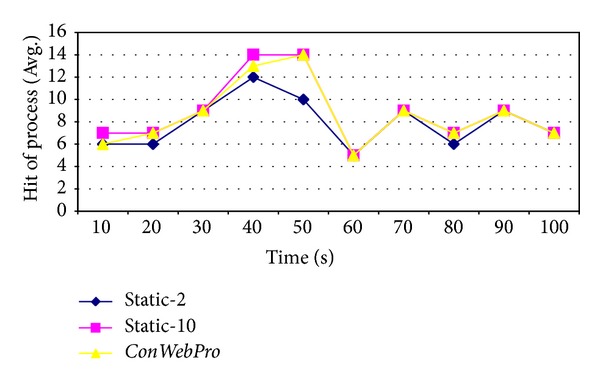
The average hit request at Gangneung-Wonju National University web traces.

Figure 6 shows the number of web processes for the web server. The *x*-axis shows the time of simulation, while the *y*-axis shows the number of times in which processes have been created for incoming requests. Following (4), a high hit rate for a process can decrease the transaction time for processing requests. We compare our* ConWebPro* scheme and two static schemes including* Static-2* and* Static-10*. Web traces from Gangneung-Wonju National University were not heavy. The average hit requests for our* ConWebPro* scheme and the* Static-10* scheme are higher than for the* Static-2* scheme; thus, the transaction time of processing request on both the* ConWebPro* and* Static-10* schemes are decreased.

**Figure 7:**
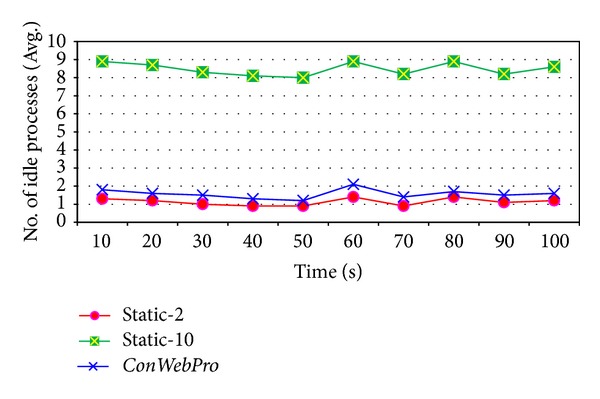
The average idle processes at Gangneung-Wonju National University web traces.

Figure 7 shows the number of idle processes for the web server. The *x*-axis shows the time of the simulation, while the *y*-axis shows the number of idle processes reserved for future requests. Following (4), the web server should maintain a small number of idle web processes to preserve the performance of web server. Even though* Static-10* increases the hit rate for incoming requests, it wastes web server resources on maintaining too many idle processes.* Static-2* shows a small number of idle processes.* ConWebPro* creates a small number of idle processes even though there is a high hit rate for web processes in Figure 6.* ConWebPro* maintains two processes exclusively for incoming requests for main objects, so* ConWebPro* has more idle processes than* Static-2*, which maintains two processes for incoming requests for all objects including main objects and embedded objects.

**Figure 8:**
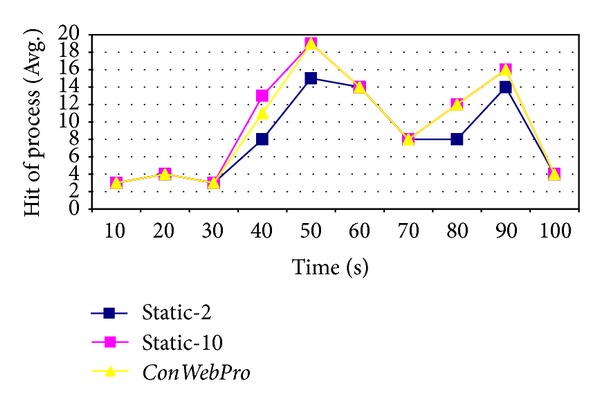
The average hit request at NASA web traces.

**Figure 9:**
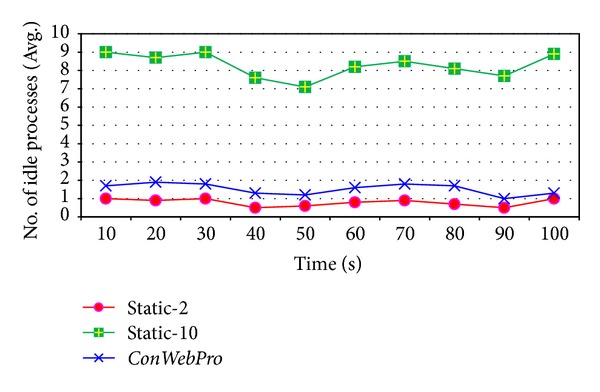
The average idle processes at NASA web traces.

Figure 8 shows the number of times processes have been created. At the initial time of simulation, incoming traffic was not heavy. Therefore, all three schemes show a similar number of hits for incoming requests. Moreover, the small number of idle processes could initially handle incoming traffic. When web traffic increased in the middle of simulation,* Static-10* and* ConWebPro* still show a high hit rate on web processes for incoming requests. However,* Static-2* increased missed processes.  Figure 9 shows how many idle processes were running on the NASA web server. At the initial time,* Static-10* created too many idle process and wasted resources. This increased overhead on the web server as shown in (4).* ConWebPro* created a small number of idle processes; therefore,* ConWebPro* conserved web server resources.

**Figure 10:**
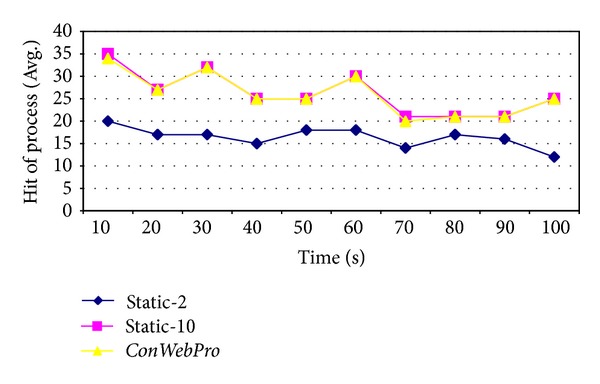
The average hit of request at Clark Net web traces.

**Figure 11:**
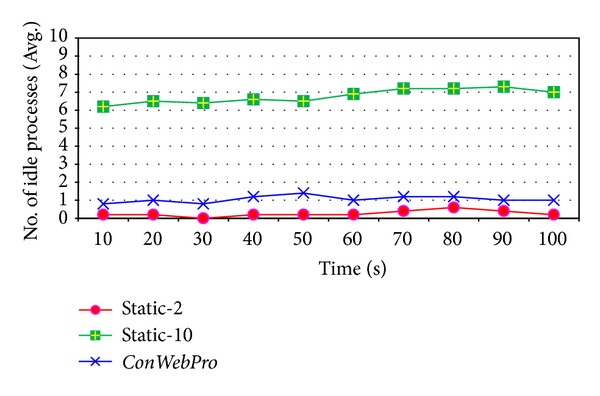
The average idle processes at Clark Net web traces.

Figure 10 shows the number of times processes had been created before incoming requests. ClarkNet contains heavy web traffic.* Static-2* shows many missed incoming requests, and* Static-10* and* ConWebPro* show a high hit rate for incoming requests.  Figure 11 shows the idle processes running on the web server.* Static-10* ran several idle processes to handle incoming requests, but it was more efficient in this case compared to light web traces like the Gangneung-Wonju National University's traffic. Therefore, we conclude that heavy web traffic is needed to justify maintaining a high number of idle processes to reduce the transaction time.* ConWebPro* can maintain the proper number of incoming requests at light and even heavy web traffic.

## 5. Conclusion

In this paper, we applied* ConWebPro* for efficient resource management on a modern web server system. To improve the quality of web service, popular web schemes including web pipeline and persistent connection consume web server system resources. Therefore, efficient resource management is critical. To be more efficient, a web server system should correctly anticipate future requests. Our* ConWebPro* predicts how many resources should be assigned to process future requests using predication based on the structure of web documents. When a web browser sends its request to a main web object containing embedded objects,* ConWebPro* adjusts idle process to handle requests for embedded objects. In future work, we will investigate how resource management affects overall performance of web server systems.

## Figures and Tables

**Table 1:** The frequency of request to the embedded objects.

Index	Name	Request
1	/atomicbk/orders.gif	63
2	/atomicbk/promo.gif	62
3	/atomicbk/catalog.gif	60
4	/atomicbk/logo2.gif	60
5	/atomicbk/shocked/shocked.jpg	60
6	/atomicbk/artgal.gif	59
7	/atomicbk/contest.gif	59
8	/atomicbk/direct.gif	58
9	/atomicbk/new.gif	57
10	/atomicbk/images/atomgirl.jpg	53
11	/atomicbk/email.gif	52
12	/atomicbk/news.gif	51
13	/atomicbk/emboss.jpg	49
14	/atomicbk/scotth.gif	49
15	/atomicbk/bobk.gif	46
16	/atomicbk/seanc.gif	44
17	/atomicbk/bizlink.gif	41
18	/atomicbk/stats.gif	34
19	/atomicbk/userlink.gif	34
20	/atomicbk/images/comic75.gif	16

**Table 2:** Web access logs.

Name	Day 1	Day 2	HTTP
CS GWNU	21559	28441	HTTP 1.1
NASA	64714	60265	HTTP 1.0
ClarkNet	210908	229944	HTTP 1.0

**Table 3:** Analysis of web objects (Gangneung-Wonju National University).

Path	Number of accesses	Number of embedded objects
/main.html	623	1
/html/main.php	359	5
/bbs/board.php	178	10
/bbs/include/md5.js	99	15
/bbs/include/script.js	77	15
/bbs/xe/common/js/jquery.min.js	65	5
/bbs/xe/common/js/jquery.min.js	64	12
/bbs/xe/common/js/xe.min.js	64	6
/bbs/xe/addons/resize image/js/resize image.min.js	64	15
/bbs/xe/modules/board/tpl/js/board.js	65	12

**Table 4:** Analysis of web objects (ClarkNet).

Path	Number of accesses	Number of embedded objects
/pub/journalism/awesome.html	1189	0
/pub/job/vk/vendela.html	772	32
/main.html	275	3
/pub/jeffd/index.html	238	7
/pub/atomicbk/catalog/sleazbk.html	271	7
/pub/sshay/interact.html	226	10
/pub/atomicbk/catalog/erotica.html	209	5
/pub/atomicbk/catalog/adultcom.html	177	10
/pub/sshay/gallery.html	186	10
/pub/job/vk/vendela2.html	142	35

**Table 5:** Analysis of web objects (NASA).

Path	Number of accesses	Number of embedded objects
/shuttle/countdown/	1043	3
/main.html	540	7
/shuttle/missions/sts-71/images/images.html	483	0
/shuttle/missions/sts-71/mission-sts-71.html	435	5
/ksc.html	420	6
/shuttle/countdown/liftoff.html	386	2
/shuttle/missions/missions.html	305	4
/history/apollo/apollo.html	147	6
/academics/courses	138	5
/shuttle/countdown/countdown.html	136	4
